# Psychological Distress Among the U.S. General Population During the COVID-19 Pandemic

**DOI:** 10.3389/fpsyt.2021.642918

**Published:** 2021-06-22

**Authors:** Christi J. Guerrini, Sophie C. Schneider, Andrew G. Guzick, Gifty N. Amos Nwankwo, Isabel Canfield, Savitri Fedson, Amanda M. Gutierrez, Jessica C. Sheu, Amber Y. Song, Alexandra M. Villagran, Amy L. McGuire, Eric A. Storch

**Affiliations:** ^1^Center for Medical Ethics and Health Policy, Baylor College of Medicine, Houston, TX, United States; ^2^Menninger Department of Psychiatry and Behavioral Sciences, Baylor College of Medicine, Houston, TX, United States; ^3^Department of Philosophy, University of Notre Dame, Notre Dame, IN, United States; ^4^Michael E. DeBakey VA Medical Center, Houston, TX, United States

**Keywords:** mental health, anxiety, depression, pandemic (COVID-19), survey

## Abstract

The COVID-19 pandemic is taking a significant global toll on emotional well-being, but evidence of mental health impacts in the United States remains limited. In April 2020, we conducted an exploratory survey of U.S. residents to understand prevalence of and factors associated with psychological distress during the pandemic. Data collection was conducted using Qualtrics, an online survey platform, and U.S. adult respondents were recruited via Amazon's Mechanical Turk platform. Among 1,366 respondents, 42% (*n* = 571) reported clinically significant anxiety and 38% (*n* = 519) reported clinically significant depression. Factors associated with anxiety and depressive symptoms included Hispanic/Latino ethnicity; younger age; lower income; employment as or living with a health care worker-first responder; caregiver status; SARS-CoV-2 infection status; decreased frequency of engagement in healthy behaviors; and changed frequency of engagement in unhealthy behaviors. That some of these factors are associated with elevated distress during the pandemic is not yet widely appreciated and might be useful in informing management of mental health care resources.

## Introduction

As the coronavirus disease 2019 (COVID-19) pandemic continues its deadly march across the globe, increased attention is being paid to associated mental health impacts. There is growing evidence that the pandemic is taking a significant toll on the emotional well-being of specific groups and general populations around the world. In studies conducted primarily in China (1), health care workers during the COVID-19 pandemic have reported clinically significant symptoms of anxiety, depression, and other mental health disorders (2–4), as they did during the severe acute respiratory syndrome (SARS) outbreak in 2003 (5–7). Other studies have found elevated psychological distress among general populations in countries around the world (8–17).

Data are also emerging about the mental health impacts of the pandemic in the United States. In a survey fielded from April 7–13, 2020, 13.6% of 1,468 respondents reported symptoms of serious psychological distress on the Kessler 6 Psychological Distress Scale (18). Prevalence of reported symptoms was highest among Hispanic/Latino respondents, respondents aged 18–29 years old, and respondents residing in households with annual income < $35,000. By comparison, in 2018, only 3.9% of U.S. adults reported serious distress on the same scale presented in the National Health Interview Survey (18). Another survey fielded from March 31 to April 13, 2020 found that 28% of respondents reported elevated depressive symptoms, which was approximately 3.5 times the prevalence identified in the 2017–2018 National Health and Nutrition Examination Survey (19). Additionally, in June 2020, 31% of 5,412 U.S. adults reported elevated anxiety or depressive symptoms on the four-item Patient Health Questionnaire (PHQ-4), with the highest prevalence reported by Hispanic/Latino respondents, respondents aged 18–24 years old, respondents who had not completed high school, and respondents who were unpaid caregivers for adults (20).

Unpublished studies of mental health concerns among U.S. residents during the pandemic include the Household Pulse Survey, fielded by the U.S. National Center for Health Statistics and Census Bureau beginning on April 23, 2020, which used modified versions of the two-item Generalized Anxiety Disorder (GAD-2) and two-item Patient Health Questionnaire (PHQ-2) scales (21). Respondents reporting clinically significant anxiety or depressive symptoms in 2020 ranged from a low of 34% in mid-May 2020 to a high of 43% in mid-November 2020, with a larger share of female respondents and respondents aged 18–29 years reporting symptoms every week. By contrast, from January–June 2019, only 11% of respondents reported these symptoms on the National Health Interview Survey, which used unmodified versions of these instruments (22).

While these data are helpful in understanding the general mental health burden of the pandemic on the U.S. population, there remains limited information regarding non-sociodemographic factors associated with elevated mental distress. These factors might relate to, for example, susceptibility to SARS-CoV-2 infection, household structure, social distancing practices, or changes to relationships or finances. Moreover, few studies have determined how individuals are engaging in self-care during the pandemic or whether those behaviors are associated with mental health. We contribute to consideration of these questions by reporting data from an exploratory online survey of U.S. residents intended to understand not only the prevalence of elevated symptoms of anxiety and depression during the early months of the COVID-19 pandemic, but also the sociodemographic, health, behavioral, and other factors associated with these symptoms. By capturing information about respondents' health, household and financial circumstances, and behaviors, as well as their self-assessments of the impacts of the pandemic on their relationships and mental health, we sought to obtain a broad understanding of their pandemic-related experiences.

## Methods

Data collection was conducted from April 17–22, 2020 using the online survey platform Qualtrics. Survey respondents were recruited via Amazon's Mechanical Turk (MTurk) platform. MTurk is an online market in which “requestors” recruit “workers” to complete tasks, including surveys (23). For a specific task, the requestor sets the eligibility criteria for workers and pays those who complete the task a predetermined amount. Between 2016 and 2018, there were at least 80,000 U.S.-based MTurk workers active each year (24). By 2016, over 1,000 papers reporting studies using MTurk, including studies of psychological symptoms and interventions, had been published in social science journals (25).

In this study, recruitment was limited to adult MTurk workers (age ≥18) who lived in the United States and had an MTurk approval rating >92% (i.e., proportion of completed tasks approved by previous requesters). Using standard MTurk procedures, a brief description of the task was published on the MTurk platform and MTurk workers who met eligibility criteria were invited to participate. Respondents who completed the survey were not allowed to participate again. The survey was eligible for waiver of the requirement for written documentation of informed consent because the risks to respondents were minimal and the study involved no procedures for which written consent is normally required outside of the research context. At the conclusion of the survey, respondents were compensated US$1.00. All survey materials were approved by the Baylor College of Medicine Institutional Review Board.

The questionnaire included six categories of items relevant to this study: distancing efforts (13 items); COVID-19 experiences (20 items); financial and social impacts (14 items); mental health impacts (28 items); anxiety symptoms using the 7-item Generalized Anxiety Disorder Scale (GAD-7) (26); and depressive symptoms using the 9-item Patient Health Questionnaire (PHQ-9) (27). Some survey items relevant to the first four categories were adapted from two questionnaires (28, 29). The GAD-7 and PHQ-9 scales are for screening purposes only and not for diagnosing syndromes. The questionnaire also included demographic items and two attention check items to identify inattentive respondents. All relevant survey items are reproduced as Supplementary Material.

A total of 1,720 responses were recorded. Responses from 345 (20.1%) participants who took <5 min to complete the survey or incorrectly answered at least one attention check item were excluded from analyses. In addition, we excluded responses from nine (0.5%) participants that provided one or more free-text responses indicating that either the respondent was inattentive or the survey was completed by a bot. The final sample for analysis consisted of 1,366 U.S.-based respondents (79.4% of the total sample). For the remainder of this report, all references to respondents means the final sample.

Statistical analysis was conducted using SPSS Version 26. *T*-tests and ANOVA were conducted to evaluate group differences in mean scores on the GAD-7 and PHQ-9. Corrected effect sizes were calculated using Hedge's *g* with cut points of 0.20 for a small effect, 0.50 for a medium effect, and 0.80 for a large effect (30). A *p*-value of < 0.001 was determined to be statistically significant.

Consistent with other studies, scores of eight or higher on the GAD-7 (31) and 10 or higher on the PHQ-9 (32) were coded as representing clinically significant symptoms. Because guidelines about risk level of activities were not available at the time of data collection, classification of activities as potentially high risk was informed by Texas Medical Association guidelines published after the survey was fielded (33).

## Results

As shown in Table 1, the majority of respondents were male (*n* = 793; 58%), White (*n* = 1,055; 77%), between 23 and 36 years old (*n* = 719; 53%), and reported annual household income of at least US$50,000 (*n* = 783; 57%). Hispanic/Latino ethnicity was reported by 17% (*n* = 234) of respondents. Most respondents (*n* = 997; 73%) were employed at the time of the survey. Among all respondents, 16% (*n* = 215) were employed in health care and 10% (*n* = 140) were employed as first responders. There was considerable overlap between health care and first responder employment: 120 of the 215 respondents employed in health care also were first responders.

**Table 1:** Respondent demographics and pandemic-related experiences (*N* = 1,366).

**Demographics**	***n* (%)**
Gender	
Male	793 (58%)
Female	563 (41%)
Other	10 (<1%)
Age	
18–22	37 (3%)
23–36	719 (53%)
37–51	399 (29%)
52–70	172 (13%)
71–88	39 (3%)
Race^a^	
White	1,055 (77%)
Native Hawaiian or other Pacific Islander	6 (<1%)
Black or African American	193 (14%)
Asian	104 (8%)
American Indian or Alaska Native	33 (2%)
Other	18 (1%)
Ethnicity	
Hispanic/Latino	234 (17%)
Employment, household, and caregiving	
Health care worker-first responder	120 (9%)
Health care worker (not first responder)	95 (7%)
Lives with health care worker-first responder	89 (7%)
Lives with health care worker (not first responder)	97 (7%)
Child or children live at home	670 (49%)
Caregiver for adult	390 (29%)
Household income	
< $10,000	44 (3%)
$10,000–$19,999	80 (6%)
$20,000–$34,999	212 (16%)
$35,000–$49,999	247 (18%)
$50,000–$74,999	352 (26%)
$75,000–$99,999	222 (16%)
$100,000–$149,999	158 (12%)
$150,000 or more	51 (4%)
**Pandemic-related experiences**	
Infection status and risk	
High likelihood infection^b^	170 (12%)
Moderate likelihood infection^c^	121 (9%)
Self at elevated risk of infection^d^	292 (21%)
Household member at elevated risk of infection^d^	332 (24%)
Financial impacts	
Lost income or employment	743 (54%)
Worse off financially	557 (41%)
Recent participation in potentially high-risk activity	
Went to a bar/nightclub	209 (15%)
Traveled by train, bus, or plane	220 (16%)
Visited church or place of worship	223 (16%)
Attended a 5+ person social gathering	241 (18%)

a *Respondents could select more than one race and so numbers add to >100%*.

b *Respondents were categorized as “high-likelihood infection” if they responded that they tested positive for infection, were told by a medical professional they had COVID-19, or were hospitalized for COVID-19*.

c *Respondents were categorized as “moderate-likelihood infection” if they responded that they had COVID-19 symptoms, believed they were infected, or had close contact with someone with confirmed infection, and otherwise did not satisfy any “high-likelihood infection” criteria*.

d *“Elevated risk” was defined consistent with Centers for Disease Control and Prevention guidelines at the time as immunocompromised, pregnant, or a current smoker; having a pre-existing respiratory disease, heart disease, or diabetes; or being over 65 years old*.

Among all respondents, 42% (*n* = 571) and 38% (*n* = 519) reported clinically significant levels of anxiety and depression, respectively. As shown in Table 2, sociodemographic factors associated with significantly higher mean symptom scores included Hispanic/Latino ethnicity. On the other hand, Asian respondents were significantly less anxious and depressed than those who self-identified as other races. Younger age was weakly associated with higher mean scores on the GAD-7 (*r* = −0.15, *p* < 0.001) and PHQ-9 (*r* = −0.17, *p* < 0.001). Significant effects of income were also found for both anxiety (*F*_(7,1358)_ = 5.18, $\eta_{partial}^{2}$=0.026, *p* < 0.001) and depression (*F*_(7,1358)_ = 7.46, η^2^_partial_ = 0.037, *p* < 0.001). Specifically, higher income was associated with lower mean anxiety (*B* = −3.39, *p* < 0.001) and depression (*B* = −4.49, *p* < 0.001) scores.

**Table 2:** Univariate *t*-tests comparing generalized anxiety and depressive symptoms across subgroups.

	**GAD-7**					**PHQ-9**				
	**$\text{M}{\left(\text{SD}\right)}_{1}^{\text{a}}$**	**$\text{M}{\left(\text{SD}\right)}_{2}^{\text{a}}$**	***g***	***t***	***P***	**$\text{M}{\left(\text{SD}\right)}_{1}^{\text{a}}$**	**$\text{M}{\left(\text{SD}\right)}_{2}^{\text{a}}$**	***g***	***t***	***P***
**Demographics**										
Male	6.70 (5.79)	7.15 (5.81)	−0.08	−1.42	0.16	7.85 (6.98)	7.99 (6.97)	−0.02	−0.35	0.72
Hispanic/Latino ethnicity	9.45 (5.88)	6.37 (5.64)	0.50^*^	7.55	<0.001	11.82 (7.31)	7.11 (6.62)	0.57^*^	9.75	<0.001
Asian	4.73 (4.39)	7.07 (5.86)	−0.41^*^	−5.08	<0.001	5.44 (5.56)	8.12 (7.04)	−0.39^*^	−4.61	<0.001
Black or African American	7.68 (5.93)	6.77 (5.77)	0.16	2.03	0.043	8.95 (7.31)	7.74 (6.90)	0.17	2.15	0.033
White^b^	7.05 (5.85)	6.42 (5.64)	0.11	1.75	0.081	8.01 (6.99)	6.63 (6.91)	0.05	0.87	0.38
**Employment and household**										
Health care worker-first responder	12.55 (4.04)	6.35 (5.65)	1.12^*^	15.41	<0.001	16.57 (4.62)	7.08 (6.58)	1.47^*^	20.57	<0.001
Health care worker (not first responder)	8.51 (6.07)	6.78 (5.75)	0.30	2.81	0.005	9.94 (7.38)	7.76 (6.92)	0.31	2.94	0.003
Lives with health care worker-first responder	11.69 (3.98)	6.56 (5.76)	0.90^*^	11.35	<0.001	15.51 (4.58)	7.39 (6.80)	1.22^*^	15.57	<0.001
Lives with health care worker (not first responder)	6.75 (5.53)	6.91 (5.82)	−0.03	−0.25	0.80	8.23 (6.79)	7.89 (6.99)	0.048	0.46	0.65
Lives alone	6.55 (6.05)	6.97 (5.75)	−0.07	−0.99	0.32	7.71 (7.49)	7.95 (6.86)	−0.03	−0.45	0.64
Lives with children	7.79 (5.94)	6.03 (5.53)	0.31^*^	5.65	<0.001	9.17 (7.45)	6.71 (6.24)	0.36^*^	6.60	<0.001
Caregiver for adult	9.95 (5.38)	5.68 (5.51)	0.78^*^	13.04	<0.001	12.31 (6.85)	6.16 (6.20)	0.96^*^	15.39	<0.001
**Health status**										
Self at elevated risk of infection^c^	8.78 (5.82)	6.39 (5.69)	0.42^*^	6.35	<0.001	10.64 (7.25)	7.17 (6.71)	0.51^*^	7.37	<0.001
Household member at elevated risk of infection^c^	8.28 (5.77)	6.45 (5.74)	0.32^*^	5.03	<0.001	9.52 (6.88)	7.40 (6.93)	0.31^*^	4.87	<0.001
High-likelihood infection^d^	12.22 (3.70)	6.14 (5.64)	1.12^*^	18.57	<0.001	15.88 (4.20)	6.78 (6.53)	1.44^*^	24.35	<0.001
Moderate-likelihood infection^e^	8.33 (5.95)	6.76 (5.77)	0.27	2.86	0.004	9.55 (7.33)	7.76 (6.92)	0.26	2.72	0.007
**Recent participation in potentially high-risk activities**										
Went to a bar/nightclub	12.18 (3.60)	5.94 (5.60)	1.17^*^	20.90	<0.001	15.88 (4.18)	6.48 (6.38)	1.54^*^	27.29	<0.001
Traveled by train, bus, or plane	11.79 (3.91)	5.96 (5.63)	1.08^*^	18.71	<0.001	15.23 (6.51)	6.51 (6.44)	1.41^*^	23.52	<0.001
Visited church or place of worship	11.56 (4.17)	5.99 (5.63)	1.03^*^	17.15	<0.001	14.95 (5.10)	6.54 (6.44)	1.35^*^	21.51	<0.001
Attended a 5+ person social gathering	11.26 (4.49)	5.96 (5.62)	0.97^*^	15.84	<0.001	14.40 (5.67)	6.53 (6.42)	1.25^*^	19.09	<0.001
**Financial and relationship impacts of pandemic**										
Lost income or employment	7.54 (5.93)	6.13 (5.55)	0.24^*^	4.51	<0.001	8.78 (7.18)	6.89 (6.57)	0.27^*^	5.07	<0.001
Worse off financially	7.12 (5.93)	6.74 (5.70)	0.07	1.20	0.23	7.47 (6.77)	8.22 (7.10)	−0.11	−1.95	0.050
Negative impact on relationship with significant other	9.82 (5.31)	5.69 (5.56)	0.75^*^	12.66	<0.001	11.54 (6.59)	6.41 (6.56)	0.78^*^	13.11	<0.001
Negative impact on other family relationships	8.80 (5.44)	5.42 (5.64)	0.61^*^	11.13	<0.001	10.34 (6.78)	6.04 (6.52)	0.65^*^	11.90	<0.001
Negative impact on social relationships	8.17 (5.60)	5.91 (5.91)	0.40^*^	7.28	<0.001	9.48 (6.78)	6.70 (6.79)	0.41^*^	7.46	<0.001
Negative impact on work relationships	8.42 (5.59)	6.27 (5.77)	0.38^*^	6.34	<0.001	9.54 (6.82)	7.25 (6.93)	0.33^*^	5.58	<0.001
	**Reference group reported more distress than comparison group**	**Reference group reported less distress than comparison group**								
No effect or weak effect (*g* < 0.20)										
Small effect (*g* ≥ 0.20–0.49)										
Moderate effect (*g* ≥ 0.50–0.79)		N/A								
Large effect (*g* ≥ 0.80–1.09)		N/A								
Very large effect (*g* ≥ 1.10)^f^		N/A								

*GAD-7 = Generalized Anxiety Disorder 7-item scale; PHQ-9 = Patient Health Questionnaire 9-item scale*.

* *p <0.001*.

a *For all comparisons, subscript 1 indicates the group references in the left column and subscript 2 indicates all others (e.g., male vs. non-male, Hispanic/Latino vs. other ethnicities, etc.)*.

b *In this comparison, “non-White” participants were classified as such if they endorsed any other race (i.e., if they identified as White and another race, they were included in the “non-White” category)*.

c *“Elevated risk” was defined consistent with Centers for Disease Control and Prevention guidelines at the time as immunocompromised, pregnant, or a current smoker; having a pre-existing respiratory disease, heart disease, or diabetes; or being over 65 years old*.

d *Respondents were categorized as “high-likelihood infection” if they responded that they tested positive for infection, were told by a medical professional they had COVID-19, or were hospitalized for COVID-19*.

e *Respondents were categorized as “moderate-likelihood infection” if they responded that they had COVID-19 symptoms, believed they were infected, or had close contact with someone with confirmed infection, and otherwise did not satisfy any “high-likelihood infection” criteria*.

f *Although Cohen (1992) did not recommend effect sizes > 1.10, it was added to this figure to aid in the interpretation of the magnitude of differences between groups*.

Table 2 also shows differences in distress according to occupational and household factors, health factors, participation in activities, and changes in relationships. Large and significant differences were found for those employed as health care worker-first responders and those living with health care worker-first responders. Further, there were large and significant differences among individuals who were caregivers for adults or had children living at home, although effect sizes were smaller for caregivers for children.

Health factors associated with anxiety and depressive symptom severity included being at high risk for SARS-CoV-2 infection or having a household member at high risk for SARS-CoV-2 infection, defined consistent with Centers for Disease Control and Prevention guidelines at the time of data collection as being immunocompromised, pregnant, or a current smoker; having a pre-existing respiratory disease, heart disease, or diabetes; or being over 65 years old. In addition, those who had very likely been infected had higher mean anxiety and depressive symptom scores than those who had not.

Elevated scores were also identified for individuals who had recently engaged in activities that we categorized as potentially high risk for infection, with the largest differences for those who reported having spent time in a bar or nightclub. Additionally, negative impacts on finances and relationships during the pandemic were associated with elevated symptomology, with small to moderate effect sizes. Those who had lost income or employment in the previous month were significantly more anxious and depressed than those who had not lost income or employment. However, changes to overall financial circumstances (taking into account investments and household members' income) were not associated with different mean symptom scores.

On the other hand, negative impacts of the pandemic on relationships were associated with higher mean scores for anxiety and depression. Elevated distress was associated with greater intimacy of the impacted relationship, with effect sizes increasing from small when a professional relationship had suffered, to approaching large when an intimate partner relationship had suffered. Relatedly, as shown in Tables 3, 4, which summarize anxiety and depression as a function of behaviors, those who had spent more quality time with family in the previous week compared to a typical pre-pandemic week were less anxious and less depressed than those who had spent less quality time with family. But the opposite was true with respect to friends: those who had spent more quality time with friends in the previous week compared to a typical pre-pandemic week had higher mean anxiety and depression scores than those who had spent less quality time with friends.

**Table 3:** Associations between behaviors and generalized anxiety (GAD-7).

	**More^a^**		**Same^a^**		**Less^a^**					
	**M (SD)**	**99% CI lower, upper**	**M (SD)**	**99% CI lower, upper**	**M (SD)**	**99% CI lower, upper**	***F***	***g*_more-less_^b^**	***g*_more-same_^b^**	***g*_more-less_^b^**
**Physical health**										
Exercised	6.34 (5.91)	5.40, 7.28	6.29 (5.56)	5.69, 6.90	8.19 (5.87)	7.40, 8.97	15.73^*^	0.008	−0.31^*^	−0.33^*^
Woke up well-rested	5.54 (5.40)	4.62, 6.45	5.54 (5.42)	4.97, 6.11	10.05 (5.43)	9.31, 10.79	99.44^*^	<0.001	−0.83^*^	−0.83^*^
Ate healthy	6.01 (5.61)	5.13, 6.90	6.01 (5.48)	5.45, 6.58	9.44 (5.85)	8.58, 10.30	48.86^*^	<0.001	−0.60^*^	−0.61^*^
Ate too much or too little	8.21 (5.59)	7.31, 9.11	5.96 (5.66)	5.41, 6.51	8.22 (5.92)	7.24, 9.21	25.94^*^	0.40^*^	−0.003	−0.39^*^
**Alcohol and drug use**										
Cigarettes	9.91 (5.67)	8.35, 11.47	6.24 (5.71)	5.77, 6.72	9.42 (5.16)	8.31, 10.53	38.04^*^	0.64^*^	0.091	−0.56^*^
3+ alcoholic drinks in 1 day	7.82 (6.06)	6.35, 9.28	6.25 (5.65)	5.76, 6.74	9.07 (5.70)	8.06, 10.08	25.67^*^	0.27	−0.22	−0.50^*^
Recreational drugs	12.96 (6.07)	9.43, 12.46	6.99 (6.65)	5.80, 6.72	13.75 (6.41)	9.38, 11.81	53.45^*^	0.90^*^	−0.13	−1.02^*^
**Emotional/spiritual**										
Meditation/prayer	6.96 (5.83)	5.95, 7.98	6.17 (5.70)	5.65, 6.70	9.83 (5.23)	8.85, 10.81	36.46^*^	0.14	−0.51^*^	−0.65^*^
Journaling	9.37 (5.68)	7.78, 10.96	6.17 (5.67)	5.70, 6.65	9.41 (5.52)	8.36, 10.46	38.66^*^	0.56^*^	−0.006	−0.57^*^
Hobby/favorite pastime	6.08 (5.61)	5.25, 6.92	6.50 (5.74)	5.90, 7.10	8.56 (5.80)	7.68, 9.44	19.09^*^	−0.073	−0.43^*^	−0.36^*^
**Interpersonal**										
Quality time with family	5.82 (5.55)	5.11, 6.52	7.26 (5.82)	6.57, 7.94	7.78 (5.89)	6.91, 8.64	13.51^*^	−0.25^*^	−0.34^*^	−0.089
Quality time with friends	8.72 (5.80)	7.24, 10.19	7.09 (5.80)	6.38, 7.80	6.48 (5.74)	5.90, 7.06	8.08^*^	0.28^*^	0.39^*^	0.11
Arguments	10.21 (5.55)	8.91, 11.51	6.24 (5.75)	5.69, 6.78	7.16 (5.57)	6.37, 7.95	29.60^*^	0.69^*^	0.55^*^	−0.16
Emotional support from others	7.64 (5.86)	6.91, 8.37	6.15 (5.58)	5.61, 6.69	10.94 (5.82)	8.90, 12.97	26.83^*^	0.26^*^	−0.56^*^	−0.85^*^
	**Reference group reported more distress than comparison group**	**Reference group reported less distress than comparison group**								
No effect or weak effect (*g* < 0.20)										
Small effect (*g* ≥ 0.20–0.49)										
Moderate effect (*g* ≥0.50–0.79)										
Large effect (*g* ≥0.80–1.09)										

*GAD-7, Generalized Anxiety Disorder 7-item scale*.

* *p <0.001*.

a *“More” indicates engaging in each behavior more often during the previous week compared to a typical pre-pandemic week, “same” indicates engaging in the behavior at the same frequency, and “less” indicates engaging in the behavior less often*.

b *Each effect size reflects the comparison between two groups (e.g., coefficients in the “g _more−same_” column reflect differences in mean anxiety symptom scores between those who engaged in each behavior more often v. the same)*.

**Table 4:** Associations between behaviors and depression (PHQ-9).

	**More^a^**		**Same^a^**		**Less^a^**					
	**M (SD)**	**99% CI lower, upper**	**M (SD)**	**99% CI lower, upper**	**M (SD)**	**99% CI lower, upper**	***F***	***g*_more-same_^b^**	***g*_more-less_^b^**	***g*_more-less_^b^**
**Physical health**										
Exercised	7.04 (6.96)	5.93, 8.15	7.18 (6.72)	6.45, 7.92	9.62 (7.05)	8.68, 7.92	19.18^*^	−0.021	−0.37^*^	−0.36^*^
Woke up well-rested	6.34 (6.67)	5.21, 7.47	6.56 (6.75)	5.85, 7.26	11.21 (6.41)	10.34, 12.08	72.78^*^	−0.032	−0.75^*^	−0.70^*^
Ate healthy	6.79 (6.78)	5.72, 7.86	6.92 (6.73)	6.23, 7.62	10.88 (6.77)	9.89, 11.88	45.79^*^	−0.019	−0.60^*^	−0.59^*^
Ate too much or too little	9.26 (6.35)	8.24, 10.28	6.76 (6.79)	6.09, 7.42	9.87 (7.44)	8.63, 11.11	27.60^*^	0.37^*^	−0.088	−0.45^*^
**Alcohol and drug use**										
Cigarettes	11.17 (6.62)	9.34, 13.00	6.96 (6.74)	6.40, 7.52	12.30 (6.46)	10.91, 13.68	58.31^*^	0.63^*^	−0.17	−0.80^*^
3+ alcoholic drinks in 1 day	8.53 (7.17)	6.80, 10.27	7.10 (6.72)	6.51, 7.68	10.96 (7.04)	9.71, 12.21	31.79^*^	0.21	−0.34	−0.57^*^
Recreational drugs	10.94 (4.66)	10.99, 14.93	6.26 (5.71)	6.46, 7.53	10.59 (4.88)	12.16, 15.34	80.59^*^	0.83^*^	0.073	−0.77^*^
**Emotional/spiritual**										
Meditation/prayer	7.40 (6.81)	6.21, 8.58	7.16 (6.84)	6.53, 7.78	11.67 (6.50)	10.45, 12.89	39.48^*^	0.035	−0.64^*^	−0.67^*^
Journaling	10.86 (7.47)	8.77, 12.95	6.90 (6.60)	6.34, 7.46	11.68 (6.94)	10.36, 13.01	50.55^*^	0.59^*^	−0.12	−0.72^*^
Hobby/favorite pastime	6.54 (6.57)	5.56, 7.51	7.74 (7.14)	7.00, 8.49	9.71 (6.66)	8.70, 10.72	19.66^*^	−0.17	−0.48^*^	−0.28^*^
**Interpersonal**										
Quality time with family	6.20 (6.44)	5.39, 7.02	8.75 (7.17)	7.90, 9.60	8.91 (6.93)	7.89, 9.93	23.66^*^	−0.37^*^	−0.41^*^	−0.023
Quality time with friends	9.91 (7.17)	8.08, 11.73	8.74 (7.24)	7.85, 9.63	7.05 (6.63)	6.38, 7.72	14.04^*^	0.16	0.43^*^	0.25^*^
Arguments	10.58 (6.39)	9.08, 12.08	7.20 (6.87)	6.55, 7.85	8.53 (7.11)	7.53, 9.53	17.94^*^	0.50^*^	0.30	−0.19^*^
Emotional support from others	8.64 (7.18)	7.74, 9.54	7.10 (6.68)	6.46, 7.74	12.98 (6.44)	10.73, 15.24	27.61^*^	0.22^*^	−0.61^*^	−0.88^*^
	**Reference group reported more distress than comparison group**	**Reference group reported less distress than comparison group**								
No effect or weak effect (*g* < 0.20)										
Small effect (*g* ≥ 0.20–0.49)										
Moderate effect (*g* ≥ 0.50–0.79)										
Large effect (*g* ≥ 0.80–1.09)										

*PHQ-9, Patient Health Questionnaire 9-Item scale*.

* *p < 0.001*.

a *“More” indicates engaging in each behavior more often during the previous week compared to a typical pre-pandemic week, “same” indicates engaging in the behavior at the same frequency, and “less” indicates engaging in the behavior less often*.

b *Each effect size reflects the comparison between two groups (e.g., coefficients in the “g _more−same_” column reflect differences in mean depressive symptom scores between those who engaged in each behavior more often v. the same)*.

Also as shown in Tables 3, 4, in general, more frequent engagement in healthy behaviors was associated with lower distress. Specifically, respondents who reported exercising, waking up feeling well-rested, eating healthy meals, participating in a hobby or favorite pastime, or meditating or praying at least as many times in the previous week compared to a typical pre-pandemic week reported lower mean anxiety and depressive symptom scores than those who engaged less frequently in those activities. Of these healthy behaviors, the score differences associated with healthy sleep behaviors were largest for both anxiety and depression.

Yet, compared to a typical pre-pandemic week, those who engaged more or less frequently in unhealthy behaviors—specifically, overeating, undereating, smoking cigarettes, or using recreational drugs—were more anxious and depressed than those who engaged in these behaviors at pre-pandemic levels. Of these unhealthy behaviors, deviation from regular drug usage was generally associated with the highest group differences in anxiety and depression. On the other hand, those who reduced the number of times per week they drank three or more alcoholic beverages in a day reported higher mean anxiety and depressive symptom scores than those who maintained alcohol consumption at pre-pandemic levels.

## Discussion

Our online survey identified clinically significant levels of anxiety or depressive symptoms among approximately 40% of 1,366 U.S.-based respondents. This finding is consistent with other U.S.-based surveys reporting clinically significant anxiety or depression among 31–43% of the U.S. population during the pandemic (20, 21).

### Race and Ethnicity

Also consistent with other studies (18–21), Hispanic/Latino, younger, and lower-income respondents reported significantly higher levels of psychiatric symptoms than non-Hispanic/Latino, older, and higher-income respondents. On the other hand, Asian respondents reported being significantly less anxious and depressed than non-Asian respondents. However, we caution against a conclusion that U.S.-based Asian populations are not experiencing poor mental health outcomes during the pandemic. Since the survey was conducted, there have been increasing reports of stigma, discrimination, and violence directed toward these populations (34, 35). For example, in one survey of over 400 Asian Americans and Asians living in the United States, 29% reported increased discrimination, 41% reported increased anxiety symptoms, and 53% reported increased depressive symptoms, where social support buffered against the negative impact of discrimination on depressive (but not anxiety) symptoms (36). A second study collecting data from March through September 2020 found a widening gap in anxiety and depressive symptoms between White Americans and Asian immigrants living in the United States beginning in May 2020 (37). However, Asian Americans experienced significantly higher anxiety and depressive symptoms than White Americans throughout the data collection period. Further research will be helpful in understanding the experiences of Asian Americans, U.S.-based Asian immigrants, and other populations with COVID-19-related stigma and discrimination and the impacts on their mental health.

### Employment, Family, and Friends

Negative impacts of the pandemic on the mental health of health care workers have been documented (2–4), and the effect of additional stressors for parents—especially working parents of young children—on their psychological well-being has been characterized (38). Our findings provide further support for the conclusion that these populations experienced clinically significant anxiety or depressive symptoms at the beginning of the pandemic. Additionally, we identified other groups that may be at elevated risk of poor mental health whose suffering has not yet received widespread attention. These neglected sufferers include health care worker-first responders, those who live with them, and adult caregivers.

Although first responders already are at risk for distress given the traumatic nature of the events to which they are regularly exposed while on duty and their potential exposure to communicable disease, the pandemic might be worsening those baseline levels. Two studies of the mental health of North American first responders unconnected to specific sentinel events identified mean scores on the GAD-7 from 4.17 to 7.51 and the PHQ-9 from 5.45 to 7.62 for populations that included firefighters, emergency medical technicians/paramedics, and call center dispatchers (39, 40). By comparison, health care worker-first responders in our sample reported a GAD-7 mean score of 12.55 and a PHQ-9 mean score of 16.57, which were the highest mean scores among all groups that we considered. Factors contributing to these outcomes merit further study but likely include trauma from frequent exposure to severely ill or recently passed COVID-19 patients and concerns about becoming infected on the job and endangering loved ones. Notably, the symptom levels of those who live with health care worker-first responders were nearly as high as those employed in this occupation. This finding is consistent with research finding elevated levels of psychological distress among friends and family members of Chinese frontline workers—defined broadly to include first responders and health care workers (13).

Also consistent with other studies (20, 41), our results indicate that adult caregivers might be disproportionately suffering during the pandemic. Anxiety and depression are known outcomes of caregiving (42), but psychological distress might be aggravated during the pandemic as a result of additional complications (such as restrictions on hospital and nursing home visitations) that caregivers face ensuring that those in their charge are safe and receiving appropriate care. Other factors that might be contributing to caregivers' worries include the vulnerability of their loved ones to SARS-CoV-2 infection and serious COVID-19 disease due to advanced age, pre-existing illness, or living conditions. Indeed, that children are at the lowest risk of severe COVID-19 illness and death compared to all other age groups might explain why mean distress scores of caregivers for adults in our survey were higher than those of caregivers for children. However, given the continued closures of many schools and daycares, anxiety and depressive symptoms of caregivers for children might be higher today than they were when the survey was fielded—perhaps especially among working mothers (43) and parents who struggle with providing at-home education (44).

While the finding of an association between spending more quality time with family members and lower mean anxiety and depressive scores was not surprising, the association between spending more quality time with friends and *higher* mean anxiety and depressive scores is difficult to explain without further research. It is possible that respondents who increased the time they spent with friends reached out to them because these respondents felt especially lonely. Or respondents might have spent more time with friends only because they were unable to spend time with family, and they experienced the reduction or absence of family support as distressing. Similar to our findings, an Italian survey conducted in April 2020 found that depressive symptoms were negatively associated with family support but did not find an association between these symptoms and friend support (45).

### Health and Health-Related Behaviors

We further found that high likelihood of past SARS-CoV-2 infection was strongly associated with clinically significant symptoms of anxiety and depression, with mean scores in the moderate to severe range. This result is concerning given the exponential increase in the infected population since the survey was fielded (46) and the persistence of symptoms and/or delayed or long-term complications of infection for “long haulers” suffering from post-acute COVID-19 (47). Future research should focus on the psychological experience of patients with both acute and post-acute COVID-19 to understand their experiences of distress and factors that contribute to heightened or prolonged distress, such as loss of income, worry about medical bills, guilt about infecting or burdening loved ones, and neuroinflammatory effects of COVID-19 illness.

Our results also suggest behaviors that could be protective against psychological distress. Consistent with other studies (48), we found that anxiety and depression were inversely correlated with frequency of engagement in healthy behaviors, including exercising, eating healthy meals, participating in hobbies or favorite pastimes, meditating or praying, and practicing good sleep hygiene. Frequency of waking up feeling well-rested was most strongly associated with positive mental health, which is noteworthy during a time when physical distancing is encouraged and personal finances might be stressed, given that one can engage in healthy sleep behaviors without interacting with others and usually at no cost. However, causation is not established; it might be that elevated distress is negatively affecting sleep quality, rather than the other way around, or that the relationship is bidirectional (49).

On the other hand, disruption of unhealthy habits, such as smoking, drinking alcohol, and using recreational drugs, was associated with higher distress, whereas maintaining unhealthy habits at pre-pandemic levels was associated with lower distress. This finding is consistent with an online survey of Dutch smokers fielded in May 2020, which identified an association between pandemic-related stress and both increased and decreased smoking (50). For some, increased intensity of engagement in unhealthy behaviors could be a response to pandemic-related stressful conditions, such as isolation or unemployment, boredom, or restricted movement. Others, however, might be cutting back on an unhealthy habit by necessity—for example, they no longer have access to or can afford the habit—or by choice—for example, they are trying to live a healthier lifestyle, perhaps to promote COVID-19 prevention or recovery. One policy implication of this finding is that temporary bans on cigarette and alcohol sales during the pandemic, which some countries have temporarily implemented at various points in time (51), might be negatively affecting the mental health of local populations even while improving their physical health, including by potentially reducing the spread of the SARS-CoV-2 virus.

Interventions might be needed to assist the large proportion of U.S. residents experiencing elevated psychological distress during the pandemic. Of particular relevance is the finding of an association between anxiety and depressive symptoms and participating in potentially high-risk activities, including visiting bars and attending places of worship. It is possible that respondents engaged in these activities in part to relieve anxiety or depression caused or exacerbated by feelings of loneliness. If so, by treating mental health concerns, interventions might have a secondary effect of reducing participation in high-risk activities and therefore the spread of disease. To support this hypothesis, however, further study of individuals' motivations for participating in potentially high-risk activities, as well as the circumstances of those activities, is needed.

### Limitations

Our study is subject to limitations. First, the survey was conducted with a non-probability sample. Although MTurk samples have been shown to be more diverse than standard Internet samples (52), our survey population was relatively young compared to the U.S. general population and the majority of respondents were male (53). Given that young adults have consistently reported higher clinically significant anxiety and depressive symptoms than older adults during the pandemic, the sample might have biased the prevalence of psychological distress that we identified. Otherwise, respondents' reported race, ethnicity, and household income were consistent with national statistics collected by the U.S. Census Bureau (53, 54).

Second, it is possible that prevalence of depression and anxiety was higher among MTurk workers compared to non-MTurk workers at the time of our survey. We are not aware of research conducted during the pandemic focused on this question, but findings of studies conducted before the pandemic are mixed. Specifically, they reported similar, higher, and lower rates of depression and anxiety among MTurk workers compared to the U.S. general population (25).

Third, individuals with preexisting psychiatric conditions were not excluded from participation, consistent with the eligibility criteria of other U.S.-based general population surveys conducted during the pandemic (18, 19, 21). It is possible that the inclusion of respondents with preexisting mental health concerns inflated mean symptom scores, although a U.S.-based survey conducted in March 2020 that restricted participation to individuals reporting no prior history of mental health conditions also found high symptomology, with 39% reporting anxiety symptoms and 19% reporting depressive symptoms for at least 3 days in the previous week (55).

Fourth, concerns have been raised about the quality of MTurk survey responses, including issues related to malingering and carelessness (25). Following best practices, however, we used MTurk reputation ratings as an eligibility condition and screened out inattentive respondents using attention check items and completion times to promote high-quality responses (56–58). Fifth, some responses might be subject to recall, social desirability, and other response biases (59). Sixth, the cross-sectional, observational nature of this study limited an ability to make causal statements related to risk factors for distress during the COVID-19 pandemic. Finally, the survey was fielded relatively early in the pandemic and respondents might answer items differently today than when they completed the survey.

## Conclusion

The COVID-19 pandemic is taking a significant global toll on emotional well-being, and it seems clear that mental health care systems will be dealing with the repercussions long after an end of the pandemic is declared. Our results are consistent with other surveys finding elevated anxiety and depression symptomology among U.S. residents and support continued investments in the broad delivery of mental health care, both in individual and group settings. Going forward, it will be important to identify which individuals are at the highest risk of distress—especially prolonged distress—and likely to benefit from prompt intervention.

## Data Availability Statement

The raw data supporting the conclusions of this article will be made available by the authors, upon reasonable request.

## Ethics Statement

This study was reviewed and approved by Baylor College of Medicine Institutional Review Board. Written informed consent for participation was not required for this study in accordance with U.S. federal and institutional requirements.

## Author Contributions

CG, AM, and ES conceived of the study and designed the survey. IC, SF, CG, AM, AS, ES, JCS, and AV contributed to critical revision of the survey and IC, AMG, CG, and AV provided support to launch and field it. AGG and SS directed data analysis. GA contributed to data analysis. CG wrote the initial draft of the manuscript. All authors contributed to its critical revision.

## Conflict of Interest

CG has received grant funding from NIH. SS has received grant funding from the NIH, Texas Higher Education Coordinating Board, Ream Foundation, and the American Red Cross. AM serves as advisor for Danaher Life Sciences, the Greenwall Foundation, Morgridge Institute for Research, and Geisinger. AM receives grant funding from NIH. ES receives book royalties from Elsevier,Wiley, Oxford, APA, Springer, and Lawrence Erlbuam. ES is a consultant for Levo Therapeutics and Biohaven. ES has received grant funding from NIH, Texas Higher Education Coordinating Board, Ream Foundation, ReBuild TX, and Greater Houston Community Foundation. The remaining authors declare that the research was conducted in the absence of any commercial or financial relationships that could be construed as a potential conflict of interest.
