# Supplementary material for: Psychological Distress Among the U.S. General Population During the COVID-19 Pandemic
Source: Front Psychiatry. 2021 Jun 22;12:642918. doi: 10.3389/fpsyt.2021.642918 (PMC8258251; doi:10.3389/fpsyt.2021.642918)
Supplement: Supplementary file 1 [file Data_Sheet_1.PDF]

Supplementary Material

Notes: The survey as conducted included skip logic. Headings were not shown to respondents.

This brief survey is being conducted as part of a research project to understand experiences related to the COVID-19 pandemic. The survey will take approximately 10 minutes to complete.

Your participation is completely voluntary and you may end the interview at any time. Your refusal to participate will involve no penalty or loss of benefits to which you might otherwise be entitled.

We will ask you some questions about your health and health behaviors, as well as the health and health behaviors of members of your household. Some of these questions might make you uncomfortable. However, we will not ask you for any personally identifying information (such as your name or email address). Your responses will be recorded by a generated identification number and kept confidential. Although your Mechanical Turk Worker ID is individually identifiable, we will only use that ID for purposes of compensation and will not link survey responses to your Worker ID.

It is possible that the data from this study might be used in future research studies without additional informed consent from you or your legally authorized representative.

There are no reasonably foreseeable benefits to you of participating in this survey, although we hope the results will be broadly useful in informing public policies related to the pandemic.

If you are willing to participate, please click the forward arrow below. Proceeding with the survey will constitute your consent to participate.

Christi J. Guerrini, JD, MPH; Amy L. McGuire, JD, PhD; Eric A. Storch, PhD; Savitri Fedson, MD
Baylor College of Medicine

Background

In late 2019, the Chinese city of Wuhan reported a novel pneumonia caused by a coronavirus named COVID-19. On March 11, 2020, the World Health Organization declared the global COVID-19 outbreak a pandemic. Since then, COVID-19 has infected many thousands of U.S. residents.

Distancing Efforts

First, we would like to understand how, if at all, the COVID-19 pandemic has affected your daily activities.

In the past week, how many times, if at all, did you leave your house?

- Never
- 1 time
- 2-4 times
- 5+ times

In the past week, how frequently, if at all, did you participate in the following activities?

	0 times	1 time	2-4 times	5+ times
Worked outside of the home				
Shopped at a store				
Ate at a restaurant or café				
Visited with someone who is not a member of your household inside your home				
Visited with someone inside their home				
Spent time in a bar or nightclub				
Spent time at a park or playground				
Spent time in a church or place of worship				
Spent the night somewhere other than your own home				

	0 times	1 time	2-4 times	5+ times
Traveled somewhere by bus, train, or airplane	○	○	○	○
Attended an in-person social gathering involving 5+ people	○	○	○	○
Attended an in-person work meeting involving 5+ people	○	○	○	○

COVID-19 Experiences

We would now like to understand your experiences and the experiences of members of your household related to COVID-19 testing and infection.

Have you had any of the following experiences related to COVID-19 testing and infection?

	Yes	No
I was tested for a COVID-19 infection	○	○
I experienced symptoms consistent with a COVID-19 infection (for example, fever, dry cough, or shortness of breath)	○	○
I was told by a medical professional that I was infected by COVID-19	○	○
I believe I was infected by COVID-19	○	○
I was hospitalized as a result of a COVID-19 infection	○	○
I had direct contact with someone who I believe was infected by COVID-19	○	○
I was instructed by a medical professional or employer to quarantine at home or in a facility	○	○

Did you test positive for a COVID-19 infection?

- ◯ Yes
- ◯ No
- ◯ I don't know - I'm waiting for the test results

How many people (including you) live in your home? Please count everyone living in your home most of the time, even if temporarily, including young children, roommates, friends, and family members.

- ◯ 1 - I live alone
- ◯ 2
- ◯ 3+

Has anyone in your household (other than you) had any of the following experiences related to COVID-19 testing and infection?

	Yes	No
They were tested for a COVID-19 infection	◯	◯
They experienced symptoms consistent with a COVID-19 infection (for example, fever, dry cough, or shortness of breath)	◯	◯
They were told by a medical professional that they were infected by COVID-19	◯	◯
I believe they were infected by COVID-19	◯	◯
They believe they were infected by COVID-19	◯	◯
They were hospitalized as a result of a COVID-19 infection	◯	◯
They had direct contact with someone who they believed was infected by COVID-19	◯	◯

	Yes	No
They were instructed by a medical professional or employer to quarantine at home or in a facility	○	○
They passed away as a result of a COVID-19 infection	○	○

Has anyone in your household (other than you) tested positive for COVID-19?

- ○ Yes
- ○ No
- ○ They don't know - they are waiting on the test results

Select the No choice below.

- ○ Yes
- ○ No

Have you or anyone in your household experienced stigma or discrimination as a result of the COVID-19 pandemic?

- ○ Yes - Please explain
- ○ No

We would now like to understand the impacts of the COVID-19 pandemic on your financial well-being and relationships.

Were you employed at any point in the past month?

- ◯ Yes
- ◯ No

In the past month, what impact, if any, has the COVID-19 pandemic had on your wages?

- ◯ My wages increased (for example, you started a new job with higher pay or worked more hours)
- ◯ My wages haven't changed
- ◯ My wages decreased (for example, you were furloughed or worked fewer hours)
- ◯ My wages were eliminated because I lost my job
- ◯ Other: ____________

How has the COVID-19 pandemic impacted your <u>overall</u> financial well-being? Please take into account impacts on your wages, the wages of other members of your household who support you, and your investments, if any, in businesses and the stock market.

- ◯ I am much better off financially
- ◯ I am slightly better off financially
- ◯ I am no better or worse off financially
- ◯ I am slightly worse off financially
- ◯ I am much worse off financially

What has been the impact of the COVID-19 pandemic, if any, on your relationships with the following people?

	Very positive impact	Slightly positive impact	No impact	Slightly negative impact	Very negative impact	Not applicable
Spouse	○	○	○	○	○	○
Partner (if not married)	○	○	○	○	○	○
Children	○	○	○	○	○	○
Parents	○	○	○	○	○	○
Siblings	○	○	○	○	○	○
Other relatives	○	○	○	○	○	○
Neighbors	○	○	○	○	○	○
Friends	○	○	○	○	○	○
Work colleagues	○	○	○	○	○	○

During the COVID-19 pandemic, how often, if at all, have your family and friends given you the emotional support that you needed?

- ○ Almost always
- ○ Sometimes
- ○ Rarely
- ○ Never

During the COVID-19 pandemic, have your family and friends given you <u>more or less</u> emotional support than they typically did before the pandemic?

- ○ More emotional support

- ◯ About the same amount of emotional support
- ◯ Less emotional support

Mental Health Impacts

We would now like to understand the mental health impacts of the COVID-19 pandemic on you and members of your household.

In the past week, how many times did you engage in the following activities <u>compared to a typical week</u> before the COVID-19 pandemic?

	More times in the past week	About the same number of times	Fewer times in the past week	Not applicable
Exercised	◯	◯	◯	◯
Woke up feeling well rested	◯	◯	◯	◯
Ate healthy meals	◯	◯	◯	◯
Ate too much or too little	◯	◯	◯	◯
Smoked cigarettes	◯	◯	◯	◯
Drank 3 or more alcoholic beverages a day	◯	◯	◯	◯
Took illegal drugs	◯	◯	◯	◯
Meditated or prayed	◯	◯	◯	◯
Wrote in a journal	◯	◯	◯	◯
Participated in a hobby or favorite pastime	◯	◯	◯	◯
Spent quality time with family	◯	◯	◯	◯
Spent quality time with friends	◯	◯	◯	◯

	More times in the past week	About the same number of times	Fewer times in the past week	Not applicable
Argued with someone	○	○	○	○

Before the COVID-19 pandemic, did you participate in treatment with a mental health professional? For example, you had regular counseling sessions with a psychiatrist, psychologist, or mental health counselor.

○ Yes
○ No

How likely are you, if at all, to seek treatment with a mental health professional to help you address distress or another mental health issue related to the COVID-19 pandemic?

○ Very likely
○ Somewhat likely
○ Somewhat unlikely
○ Very unlikely

Have you been able to continue treatment with a mental health professional during the COVID-19 pandemic?

○ Yes - I have continued treatment as usual
○ For the most part - I have continued treatment but have experienced some disruptions
○ No - I have had to stop treatment

How has your treatment with a mental health professional been disrupted during the COVID-19 pandemic?

In what format have you continued treatment with a mental health professional during the COVID-19 pandemic? Please select all that apply.

- [ ] In person
- [ ] On the telephone
- [ ] By videoconference
- [ ] Other:

Have you increased the frequency of your treatment with a mental health professional during the COVID-19 pandemic? For example, you increased the number of weekly counseling sessions from 1 to 2.

- ○ Yes
- ○ No but I want to
- ○ No and I don't want to

How likely are you, if at all, to continue treatment with a mental health professional after the COVID-19 pandemic has ended?

- ○ Very likely
- ○ Somewhat likely

- Somewhat unlikely
- Very unlikely

How likely are you, if at all, to increase the frequency of your treatment with a mental health professional after the COVID-19 pandemic has ended?

- Very likely
- Somewhat likely
- Somewhat unlikely
- Very unlikely

Before the COVID-19 pandemic, did you regularly take prescription medication for distress or a mental health issue? For example, medication to address anxiety, depression, or obsessive-compulsive disorder.

- Yes
- No

How likely are you, if at all, to seek prescription medication to help you address distress or a mental health issue related to the COVID-19 pandemic?

- Very likely
- Somewhat likely
- Somewhat unlikely
- Very unlikely

Have you been able to continue taking your prescription medication for distress or a mental health issue during the COVID-19 pandemic?

- ◯ Yes - I have been able to take my medication as usual
- ◯ For the most part - I have been able to take my medication but have experienced some disruptions in my ability to do so
- ◯ No - I have had to stop taking my medication

How has your ability to take prescription medication for distress or a mental health issue been disrupted during the COVID-19 pandemic?

Have you increased the dosage of any prescription medication for distress or a mental health issue during the COVID-19 pandemic?

- ◯ Yes
- ◯ No but I want to
- ◯ No and I don't want to

How likely are you, if at all, to continue taking prescription medication to address distress or a mental health issue after the COVID-19 pandemic has ended?

- ◯ Very likely
- ◯ Somewhat likely
- ◯ Somewhat unlikely
- ◯ Very unlikely

How likely are you, if at all, to increase the dosage of any prescription medication that you take for distress or a mental health issue after the COVID-19 pandemic has ended?

- Very likely
- Somewhat likely
- Somewhat unlikely
- Very unlikely

Choose the number 5 below.

- 2
- 5
- 11
- 20

GAD-7

Over the last 2 weeks, how often have you been bothered by the following problems?

	Not at all	Several days	Over half the days	Nearly every day
Feeling nervous, anxious, or on edge	◯	◯	◯	◯
Not being able to stop or control worrying	◯	◯	◯	◯
Worrying too much about different things	◯	◯	◯	◯
Trouble relaxing	◯	◯	◯	◯
Being so restless that it's hard to sit still	◯	◯	◯	◯
Becoming easily annoyed or irritable	◯	◯	◯	◯
Feeling afraid as if something awful might happen	◯	◯	◯	◯

If you checked off any problems, how difficult have these made it for you to do your work, take care of things at home, or get along with other people?

- ◯ Not difficult at all
- ◯ Somewhat difficult
- ◯ Very difficult
- ◯ Extremely difficult

PHQ-9

We will now ask you about your current mental and emotional well-being.

Over the past 2 weeks, how often have you been bothered by any of the following problems?

	Not at all	Several days	More than half the days	Nearly every day
Little interest or pleasure in doing things	◯	◯	◯	◯
Feeling down, depressed, or hopeless	◯	◯	◯	◯
Trouble falling asleep, staying asleep, or sleeping too much	◯	◯	◯	◯
Feeling tired or having little energy	◯	◯	◯	◯
Poor appetite or overeating	◯	◯	◯	◯
Feeling bad about yourself - or that you're a failure or have let yourself or your family down	◯	◯	◯	◯
Trouble concentrating on things, such as reading the newspaper or watching television	◯	◯	◯	◯

	Not at all	Several days	More than half the days	Nearly every day
Moving or speaking so slowly that other people could have noticed. Or, the opposite - being so fidgety or restless that you have been moving around a lot more than usual	○	○	○	○
Thoughts that you would be better off dead or of hurting yourself in some way	○	○	○	○

Demographics

How do you describe your gender?

- ◯ Male
- ◯ Female
- ◯ I don't identify as male or female
- ◯ I prefer not to answer

How old are you?

- ◯ 18-22
- ◯ 23-36
- ◯ 37-51
- ◯ 52-70
- ◯ 71-88
- ◯ 89 or older

Are you of Hispanic, Latino, or Spanish origin?

- ◯ Yes
- ◯ No

How do you describe your race? Please select all that apply.

- ☐ American Indian or Alaska Native
- ☐ Asian
- ☐ Black or African American
- ☐ Native Hawaiian or Other Pacific Islander

- [ ] White
- [ ] Other:

What was your total household income (before taxes) from all sources in the last year?

- ○ Less than $10,000
- ○ $10,000 - $19,999
- ○ $20,000 - $34,999
- ○ $35,000 - $49,999
- ○ $50,000 - $74,999
- ○ $75,000 - $99,999
- ○ $100,000 - $149,999
- ○ $150,000 or more

Please select the button on this scale that best indicates your political orientation.

- ○ Liberal
- ○
- ○
- ○
- ○
- ○ Moderate
- ○
- ○
- ○
- ○
- ○ Conservative

On a scale of 1 to 5, how important is religion or spirituality in your life?

- 1 - Not at all important
- 2
- 3
- 4
- 5 - Very important

Do you have children that currently live at home with you (full-time or part-time)?

- Yes
- No

Are you a caregiver for anyone other than children? For example, a spouse or elderly parent with a health condition.

- Yes - I am a full-time caregiver
- Yes - I am a part-time caregiver
- No

Are you currently employed in health care? For example, you are a physician, emergency medical technician (EMT), or hospital janitorial worker.

- Yes
- No

Are you currently employed as a first-responder? For example, you are a police officer or firefighter.

- ◯ Yes
- ◯ No

As part of your job, do you currently have direct contact with patients infected by COVID-19?

- ◯ Yes
- ◯ No

Are you at elevated risk for serious illness if you are infected by COVID-19? You are at elevated risk if you are immunocompromised, have a pre-existing respiratory disease, have heart disease, have diabetes, are pregnant, are a current smoker, or are over 65 years old.

- ◯ Yes
- ◯ No

Is anyone in your household (other than you) currently employed in health care? For example, they are a physician, emergency medical technician (EMT), or hospital janitorial worker.

- ◯ Yes
- ◯ No

Is anyone in your household (other than you) currently employed as a first-responder? For example, they are a police officer or firefighter.

- Yes
- No

As part of their job, does anyone in your household (other than you) currently have direct contact with patients infected by COVID-19?

- Yes
- No

Is anyone in your household (other than you) at elevated risk for serious illness if they are infected by COVID-19? They are at elevated risk if they are immunocompromised, have a pre-existing respiratory disease, have heart disease, have diabetes, are pregnant, are a current smoker, or are over 65 years old.

- Yes
- No
